# Supplementary material for: Optical Microscopy-Guided Laser Ablation Electrospray Ionization Ion Mobility Mass Spectrometry: Ambient Single Cell Metabolomics with Increased Confidence in Molecular Identification
Source: Metabolites. 2021 Mar 27;11(4):200. doi: 10.3390/metabo11040200 (PMC8065410; doi:10.3390/metabo11040200)
Supplement: Supplementary file 1 [file metabolites-11-00200-s001.pdf]

Supplementary information for:

Optical microscopy-guided laser ablation electrospray ionization ion mobility mass spectrometry: ambient single cell metabolomics with increased confidence in molecular identification

Michael J. Taylor[1], Sara Mattson[2], Andrey Liyu[1], Sylwia A. Stopka[2,3], Yehia Ibrahim[1], Akos Vertes[2], and Christopher R. Anderton*[1]

[1] Earth and Biological Sciences Directorate, Pacific Northwest National Laboratory, Richland, WA, USA 99352
[2] Department of Chemistry, W. M. Keck Institute for Proteomics Technology and Applications, The George Washington University, Washington, DC 20052
[3]Department of Neurosurgery, Brigham and Women's Hospital, Harvard Medical School, Boston, MA 02115

*Christopher.Anderton@pnnl.gov; 902 Battelle Boulevard, Richland, Washington 99352; 509-371-7970

Table of Figures

Figure S1: Determining the sensitivity of the LAESI-DTIMS microscope with verapamil ……………………….…………2

Figure S2: Technical noise from ablation of droplets of standards ……………………………………………….........…2

Figure S3: Images of laser spots on *ZAP-IT* paper in beam focusing experiments over a range of Z stage heights………..4

Figure S4: The effect of sample stage height on spot uniformity and spot size ……………….………..………………..…..3

Figure S5: Large area stitched image mosaic of a A. Cepa tissue section…………………………………………………….3

Table S1: Raw counts from saccharides detected in 60 cell dataset……………………………………………..…..4

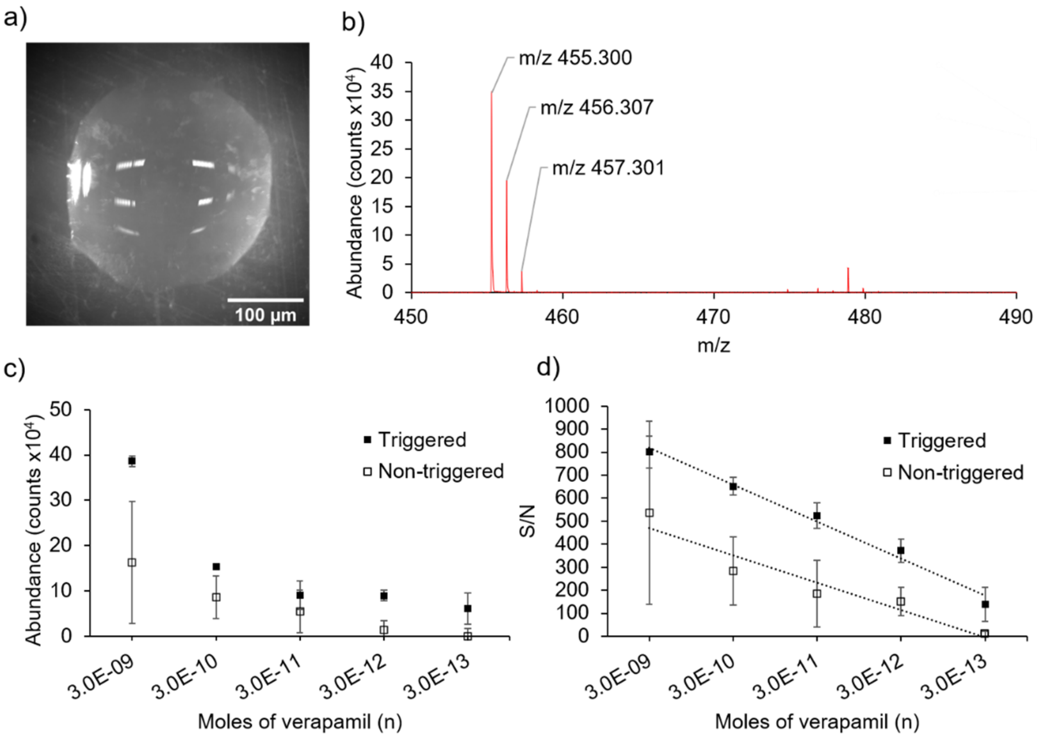

Figure S1. Determining the sensitivity of the LAESI-DTIMS microscope for detection of verapamil. a) Brightfield image of a verapamil solution droplet on the sample surface taken from the optical microscope component of the system, b) example positive ion mode verapamil spectrum, with its distinct peaks at *m/z* 455.300 $[M+H]^+$ its isotopic envelope at m/z 456.307, and m/z 457.301. c) Comparison of MS triggered (■) and MS non-triggered (□) laser firing on sensitivity and ion abundance variability of verapamil standards at different concentrations (1.0 x 10^{-3} to 1.0 x 10^{-7} mol/l), error bars are shown as standard deviation, n=10. d) Linear regression showing the limit of detection for MS triggered (calculated to be 32 fmol) and MS non-triggered (calculated to be 7,460 fmol) sampling for verapamil standards. These results show that MS-triggered mode is more sensitive than non-triggered mode.

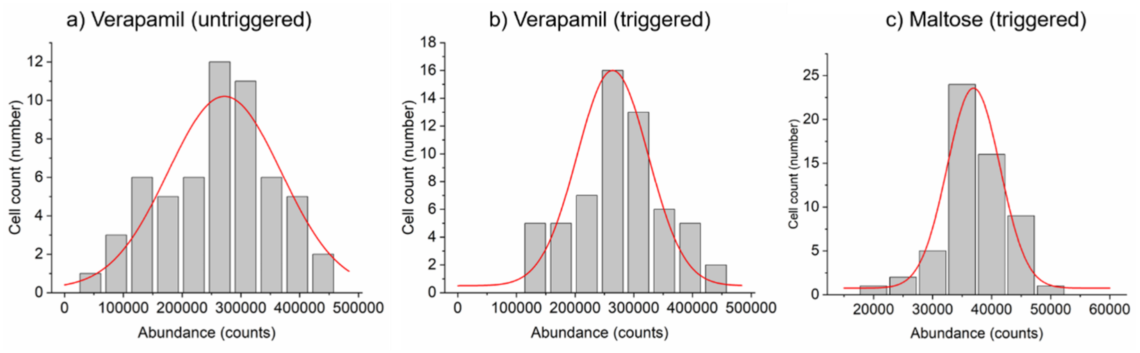

Figure S2. Technical noise from ablation of droplets of standards (3 µl of 1.0 x 10^{-3} mol/l) using non-triggered (verapamil) and triggered (verapamil, maltose) with the LAESI-IMS-MS molecular microscope. Gaussian fitting was performed in Origin, n = 10. These results show that timing the laser firing with the ion accumulation window of the DTIMS system minimizes detection variance.

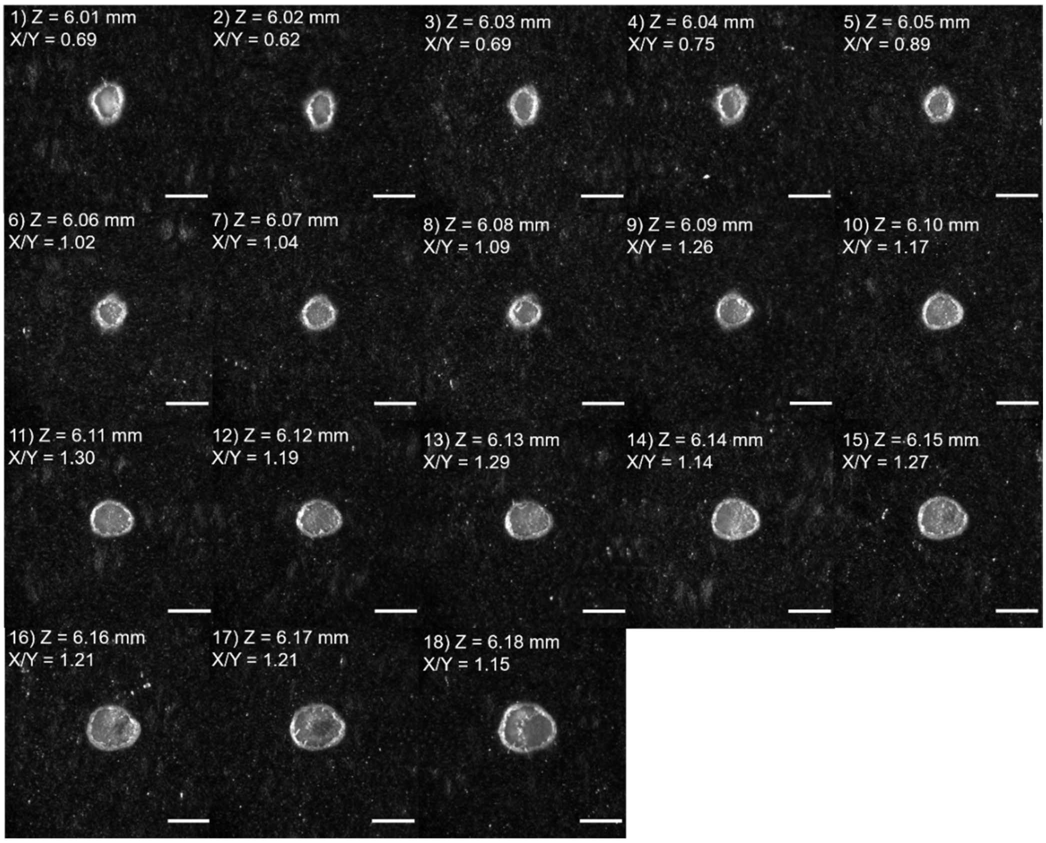

Figure S3. (1-18) Images of laser spots on ZAP-IT paper in beam focusing experiments over a range of Z stage heights defined as distance from the capillary inlet to the sample surface (mm). A single laser shot per measurement was fired for each image with a laser power of ~1.065 mJ. Scale bars shown are 100 µm. These results show that increasing the distance from the sample surface to the MS inlet and laser objective defocuses the laser, the optimal focus is tile 6), Z distance of 6.06 mm, spot diameter of 52.06 µm.

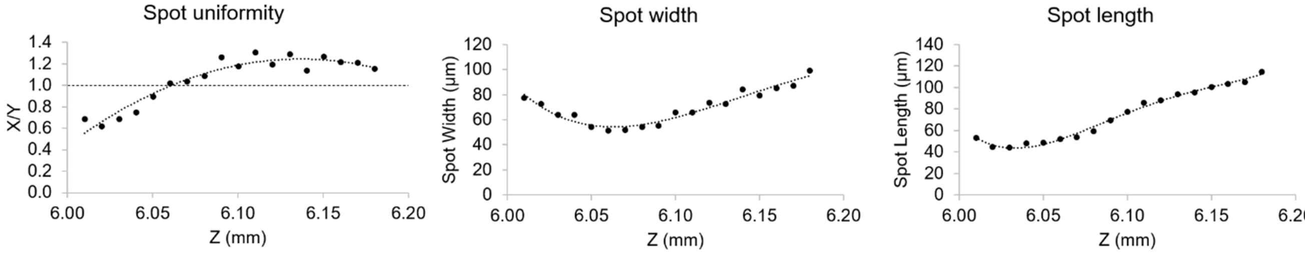

Figure S4. The effect of sample stage height on spot size and uniformity demonstrated with ZAP-IT paper based upon data shown in Figure S4. Laser spot uniformity (left graph). Y axis displays ratio of spot size width/length (µm), X axis dispalys Z stage height (µm) given as distance from sample surface to MS capillary inlet. Intercept of line of best fit with dashed line equals best laser focus, i.e. (width/length = 1). Laser spot size (middle, right graph) over a range of Z heights, Y axis displays spot length or width (µm), X axis displays Z stage height (µm) given as distance from sample surface to MS capillary inlet. These results show the optimal spot size for cell analysis is a Z height of 6.06 mm.

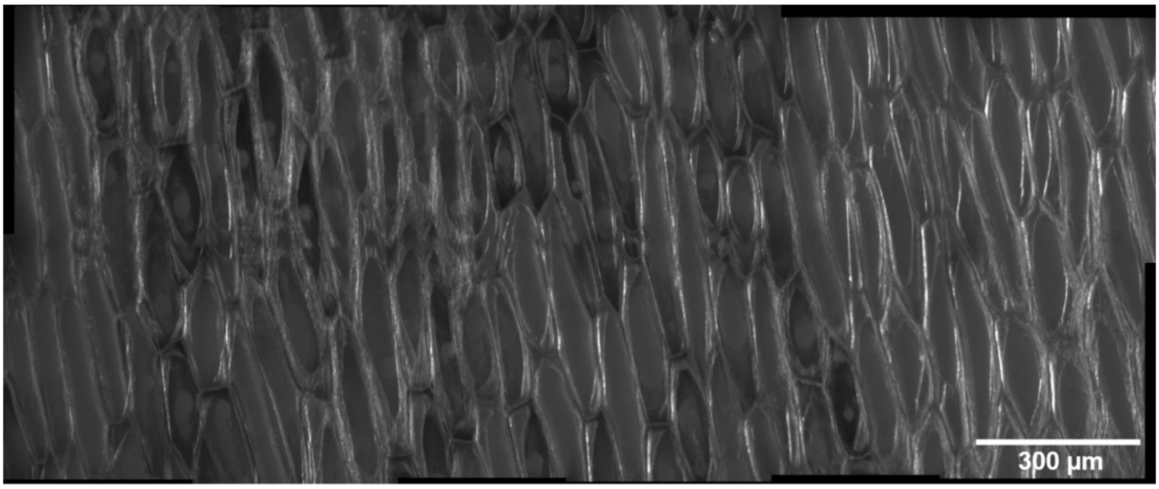

Figure S5. Large area stitched image mosaic of a *A. Cepa* tissue section. The 12 separate image tiles are recombined into a 6 x 2 (X/Y) mosaic based on feature recognition. This image shows that image tiles can be recombined into a large area mosaic using our workflow.

Table S1. Raw counts of different saccharide species detected in 60 *A. Cepa* cells. number of repeat units (r).

	**Saccharide raw counts in 60 cell dataset**			
**Cell number (no.)**	**Monosaccharide** (r = 1)	**Disaccharide** (r = 2)	**Trisaccharide** (r = 3)	**Tetrasaccharide** (r = 4)
**1**	96128	64607	14850	8152
**2**	79206	61679	21363	4621
**3**	39387	45590	22939	6090
**4**	29348	63223	17114	7475
**5**	96085	64612	12773	7502
**6**	95285	54607	12955	2235
**7**	61024	61672	23483	2394
**8**	26708	28480	18145	5564
**9**	73118	73223	10153	7069
**10**	80001	72185	19805	6767
**11**	54384	31870	23450	4316
**12**	61319	45994	19013	4759
**13**	1702	40994	15846	4304
**14**	1551	41994	16332	2653
**15**	3039	45862	21431	3324
**16**	4042	46481	20096	3193
**17**	1698	61675	21260	4975
**18**	80024	62185	19124	7975
**19**	2733	45906	17956	3664
**20**	2265	45024	21257	8441
**21**	8137	40989	18207	892
**22**	8459	45820	16195	5849
**23**	1731	45991	15028	5907
**24**	45174	46472	12787	4202
**25**	87277	45691	14544	1807
**26**	29401	41024	18100	5840
**27**	95181	32607	13542	4042
**28**	87273	41029	14525	8371
**29**	73102	73024	12872	4048
**30**	79911	62182	16561	3734
**31**	87342	31865	1049	3893
**32**	61327	45981	19843	5978
**33**	12773	62214	11062	6077
**34**	12767	38214	18348	4249
**35**	21996	20386	21000	5396
**36**	13603	34149	21648	6352
**37**	21862	65634	15197	8138
**38**	18389	32214	19942	7604
**39**	2201	55634	18830	7138
**40**	62024	19670	14759	3064
**41**	18421	14430	15693	5594
**42**	82963	39974	16591	5820
**43**	68707	31990	16464	3504
**44**	74901	55929	19545	1884
**45**	50535	30008	17665	5972
**46**	57612	42727	15288	7111
**47**	12678	31406	14590	7684
**48**	1200	49714	20848	5362
**49**	12062	44430	13763	9181

**50**	13064	50902	16982	6481
**51**	19935	62597	17636	1512
**52**	17703	41792	16438	5065
**53**	95125	64617	16084	5747
**54**	9623	54526	12834	3703
**55**	11024	41406	21361	7073
**56**	3137	45585	1407`	8348
**57**	1558	70990	19756	2636
**58**	20914	49670	19794	4616
**59**	74821	45929	16387	1380
**60**	3131	50920	14081	3736
